# Retrospective study from a single center to comparison of diagnostic value of three thyroid imaging reporting and data systems in Bethesda III/IV thyroid nodules

**DOI:** 10.3389/fonc.2025.1549646

**Published:** 2025-04-11

**Authors:** Jie Guo, Liang Du, Wenjuan Bi, Yuchen Liu, Cuiming Zhang

**Affiliations:** ^1^ College of Medical Imaging, Shanxi Medical University, Taiyuan, Shanxi, China; ^2^ The Second Hospital of Shanxi Medical University, Taiyuan, Shanxi, China; ^3^ Pingshan Hospital, Southern Medical University, Shenzhen, Guangdong, China

**Keywords:** Thyroid Imaging Reporting and Data System (TI-RADS), cytological diagnosis, Bethesda III/IV, thyroid nodules, malignancy

## Abstract

**Objectives:**

To compare the diagnostic value of ACR Thyroid Imaging Reporting and Data System (TI-RADS), K-TIRADS, and C-TIRADS in Bethesda III/IV thyroid nodules.

**Methods:**

This single-center retrospective study classified 80 Bethesda stage III/IV thyroid nodules from 80 patients between January 2020 and July 2023 according to three different systems. Diagnostic performance was evaluated using receiver operating characteristic curves, with histopathological diagnosis serving as the reference standard.

**Results:**

Overall, 41/80 (51.2%) nodules were malignant and 39/80 (48.8%) were benign. The malignancy rates for Bethesda type III and IV nodules were 50.7% and 55.6%, respectively. The malignancy risk in thyroid nodules increased with higher TI-RADS categories (*P*<0.001). Optimal cutoff values for ACR-, K-, and C-TIRADS were categories 5, 5, and 4C, respectively. Area under the curve (AUC) for ACR-, K-, and C-TIRADS was 0.782, 0.767, and 0.842, respectively, with C-TIRADS showing a significantly higher AUC than ACR-TIRADS and K-TIRADS (all *P*<0.05). C-TIRADS demonstrated the highest sensitivity, accuracy, and positive predictive value, whereas ACR TI-RADS showed the highest specificity and negative predictive value. Furthermore, the AUC, sensitivity, specificity, and accuracy of ACR TI-RADS, K-TIRADS, and C-TIRADS were higher in nodules >1 cm than in those ≤ 1 cm.”

**Conclusion:**

All three TI-RADS systems have diagnostic value in differentiating benign from malignant Bethesda III/IV nodules, With C-TIRADS showing the highest area under the curve(AUC), suggesting its potential utility in clinical evaluation and management of such nodules, particularly in Chinese populations.

## 1 Introduction

Thyroid nodules are relatively common in clinical practice, with approximately 5% nodules being malignant (1). Fine-needle aspiration (FNA) is the primary clinical method for preoperatively assessing whether thyroid nodules are benign or malignant. However, cytologically, about 20–30% nodules are classified as nodules of indeterminate significance (Bethesda categories III and IV), with a variable risk of malignancy (ROM) (2). Ultrasound is the preferred imaging modality for assessing the ROM of thyroid nodules and can effectively determine whether a nodule warrants FNA. Since Horvath first introduced the Thyroid Imaging Reporting and Data System (TI-RADS) (3)in 2009, different ultrasound classification systems for thyroid nodules have been proposed by various scholars and organizations worldwide. Among the most notable are the ACR-TIRADS, proposed by the American College of Radiology in 2017, which employs a classification assignment method (4); the K-TIRADS, revised by the Korean Society of Thyroid Radiology in 2020, based on internal structure, echogenicity, and other ultrasound features of thyroid nodules (5); and the C-TIRADS, proposed by the Chinese Society of Ultrasound in Medicine in 2020, which stratifies malignancy risk based on one benign and five malignant features of thyroid nodules (6). Despite the availability of these systems, which TI-RADS offers superior diagnostic performance for nodules with indeterminate cytology, particularly when considering different nodule sizes, thereby guiding better clinical decision-making remains unclear. This study applied these three TI-RADS classifications to thyroid nodules that were categorized using FNA cytology as Bethesda categories III/IV. The primary aim was to compare the diagnostic value of ACR-, K-, and C-TIRADS to distinguish between malignant and benign nodules.

## 2 Materials and methods

### 2.1 Patients

A retrospective analysis was conducted on 2,464 thyroid nodules that underwent FNA biopsy at the Second Hospital of Shanxi Medical University between July 2017 and September 2023. Among these, 256 nodules were classified as Bethesda category III/IV, and 80 nodules from 80 patients were included in the study. The inclusion criteria were: 1) complete clinical and thyroid ultrasound examination data; 2) cytological diagnosis of Bethesda category III/IV nodules; and 3) postoperative histopathological diagnosis. The study excluded patients: 1) who did not undergo preoperative thyroid ultrasound or whose ultrasound images were unsuitable for analysis and; 2) whose pathological diagnosis was unclear after operation. This study was approved by the Medical Ethics Committee of the Second Hospital of Shanxi Medical University. The requirement for informed consent was waived due to the retrospective nature of the study.

### 2.2 Ultrasound examination and TI-RADS classification

The ultrasound diagnostic equipment used for routine examinations was the Phillips Epiq7, equipped with a high-frequency linear array probe operating at 5–14 MHz. For FNA, an eL18-4 broadband linear array probe with a frequency of 15 MHz was used. The thyroid ultrasound images were independently reviewed retrospectively by two sonographers with over five years of experience in thyroid ultrasound examinations. The experts conducted the analysis at the same reporting workstation while being blinded to the patients’ clinical information or surgical pathology results to ensure objectivity. Before the assessment, the two experts extensively reviewed the relevant literature and guidelines on the three thyroid ultrasound classification criteria. Subsequently, they analyzed the ultrasound features of 80 thyroid nodules and classified them according to ACR-, K-, and C-TIRADS classification systems. In cases of diagnostic disagreement, a senior chief physician evaluated and determined the classification.

### 2.3 Statistical analysis

Statistical analysis was performed using SPSS 26.0 software. Normally distributed continuous data were expressed as mean ± standard deviation, and comparisons between groups were made using the t-test. In contrast, non-normally distributed continuous data were represented as median (interquartile range) [M (P_25_, P_75_)] and analyzed using the non-parametric Mann–Whitney U test. Categorical data were presented as frequency (percentage) [n (%)] and compared using either chi-square test or Fisher’s exact test, depending on the data characteristics. To assess the linear trend of TI-RADS in Bethesda III/IV nodules, Cochran–Armitage trend test was employed. Receiver operating characteristic curves were plotted to evaluate the diagnostic performance of the three TI-RADS, with the DeLong test used to compare the ROC curves of the three TI-RADS, and the differences in AUC along with their 95% confidence intervals were calculated. *P*-value < 0.05 was considered statistically significant.

## 3 Results

### 3.1 Clinicopathological characteristics

Eighty thyroid nodules from 80 patients were included in the study. Among these, 71 were classified as Bethesda III (88.8%) and nine as Bethesda IV (21.2%). Postoperative histopathological analysis confirmed that 41 nodules (51.2%) were malignant, comprising 32 cases of papillary thyroid carcinomas and nine cases of Microcarcinomas. Conversely, 39 nodules (48.8%) were benign, including 27 cases of nodular goiter, five of follicular adenoma, two of chronic lymphocytic thyroiditis, one of Hürthle cell adenoma, one of subacute thyroiditis, one of diffuse toxic goiter with adenomatous nodule, and two cases of Hashimoto’s thyroiditis. Specifically, among the Bethesda III nodules, 36 (50.7%) were malignant, and 35 (49.3%) were benign. In Bethesda category IV, five nodules (55.6%) were malignant, while four (44.4%) were benign. The malignancy rates for both categories in this study exceeded the ROM recommended by the Bethesda System for Reporting Thyroid Cytopathology (TBSRTC) (**Table 1**).

**Table 1:** Malignancy rates of Bethesda categories III/IV.

Bethesda category	Benign	Malignant	Malignancy rate (%)	Recommended malignancy rates (%)*
III	35	36	50.7	6–18
IV	4	5	55.6	10–40

*The 2017 Bethesda System for Reporting Thyroid Cytopathology (Recommended risk of malignancy in noninvasive follicular thyroid tumors with papillary features).

### 3.2 Clinical and ultrasonographic features

A comparison of clinical and ultrasound features between benign and malignant nodules revealed no statistically significant difference in patient age, gender, thyroid-stimulating hormone (TSH) levels, and nodule location (*P*>0.05). However, significant differences were observed in nodule composition, echogenicity, margins, aspect ratio, calcification type, and nodule size (*P*<0.05) (**Table 2**).

**Table 2:** Clinical and ultrasound characteristics of Bethesda categories III/IV.

Parameter	Malignant	Benign	t/Z/X^2^ value	*P*-value
Mean age (years)	46.9 ± 11.0	47.8 ± 12.2	0.353	0.725
TSH (uIU/mL)#	1.15 (0.70, 2.37)	1.14 (0.66, 1.68)	−0.789	0.430
Sex, n (%)			0.478	0.489
Males	6 (14.6)	8 (20.5)		
Females	35 (85.4)	31 (79.5)		
Location, n (%) *				0.502
Left	18 (43.9)	21 (53.8)		
Right	22 (53.7)	18 (46.2)		
Isthmus	1 (2.4)	0 (0.0)		
Size (mm), n (%)			4.033	0.045
≤1 cm	27 (65.9)	17 (43.6)		
>1 cm	14 (34.1)	22 (56.4)		
Composition, n (%)				
Solid-cystic	2 (4.9)	9 (23.1)		
Solid	39 (95.1)	30 (76.9)		
Echogenicity, n (%) *				<0.001
Hyperechoic/isoechoic	1 (2.4)	17 (43.6)		
Hypoechoic	35 (85.4)	21 (53.8)		
Markedly hypoechoic/	5 (12.2)	1 (2.6)		
Margin, n (%)			7.376	0.025
Smooth	7 (17.1)	17 (43.6)		
Ill-defined	21 (51.2)	16 (41.0)		
Irregular	13 (31.7)	6 (15.4)		
calcification, n (%) *				0.004
No echogenic foci	11 (26.8)	19 (48.7)		
Macrocalcifications	9 (22.0)	13 (33.3)		
Microcalcification	20 (48.8)	5 (12.8)		
Peripheral calcifications	1 (2.4)	2 (5.1)		
Aspect ratio, n (%)			4.089	0.043
<1	28 (68.3)	34 (87.2)		
>1	13 (31.7)	5 (12.8)		

#Expressed as “M (P25, P75),” using Mann–Whitney rank sum test.

*Using Fisher’s exact probability test.

### 3.3 Malignancy risk across the three TI-RADS

Statistical analysis revealed significant differences in ROM across the three TI-RADS, with all showing a marked increase in risk as stratification levels rose (*P* for tend^2^ < 0.001). Specifically, the ROMs for ACR-TIRADS categories 3, 4, and 5 were 0.0% (0/0), 29% (9/31), and 76.2% (32/42), respectively (*P* for tend^2^ <0.001). Similarly, for K-TIRADS categories 3, 4, and 5, the ROMs were 0.0% (0/0), 35.1% (13/37), and 77.8% (28/36) (*P* for tend^2^ <0.001). For C-TIRADS, the ROMs for categories 4a, 4b, 4c, and 5 were 15.0% (3/20), 42.1% (8/19), 82.4% (28/34), and 100% (2/2), respectively (*P* for tend^2^ <0.001) (**Table 3**).

**Table 3:** Malignancy risk of Bethesda category III/IV thyroid nodules in different TI-RADS.

Category	Benign (n)	Malignant (n)	Malignancy rate (%)	Recommended malignancy rate (%)	*P*-value	*P* for trend^2^
ACR-TIRADS					<0.001	<0.001
3	7	0	0.0	5%		
4	22	9	29.0	5%–20%		
5	10	32	76.2	>20%		
K-TIRADS					<0.001	<0.001
3	7	0	0.0	3%–10%		
4	24	13	35.1	10%–40%		
5	8	28	77.8	>60%		
C-TIRADS					<0.001	<0.001
3	5	0	0.0	<2%		
4A	17	3	15.0	2%–10%		
4B	11	8	42.1	10%–50%		
4C	6	28	82.4	50%–90%		
5	0	2	100	>90%		

### 3.4 Diagnostic value of the three TI-RADS

**Figure 1:**
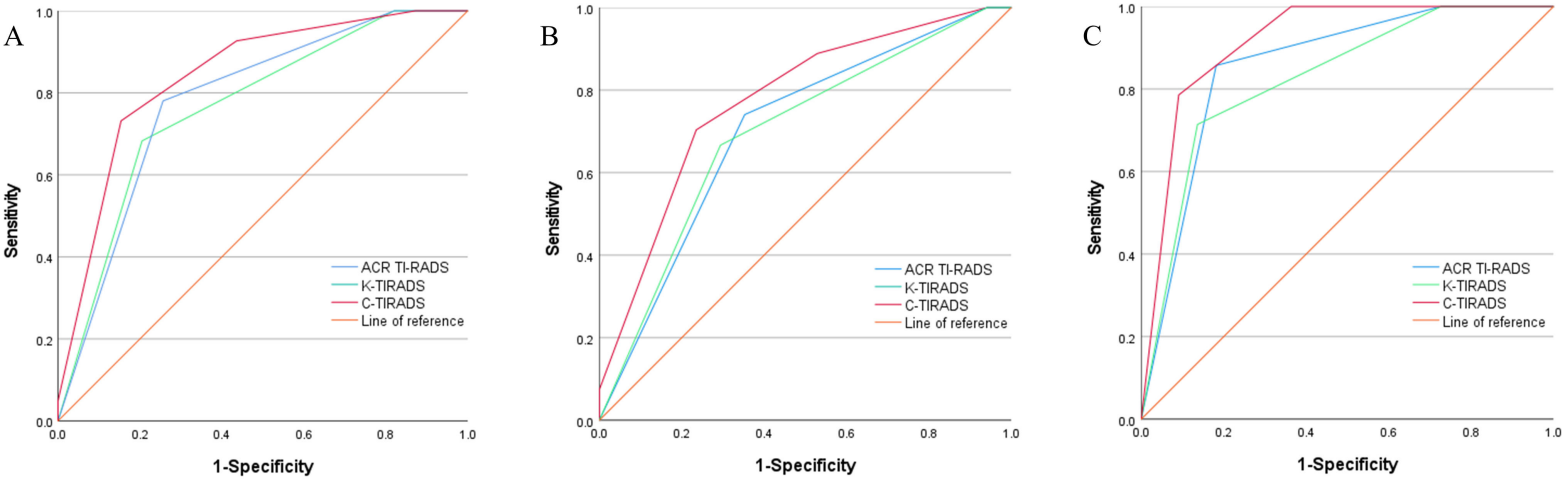
Receiver operating characteristic curves for ACR-TIRADS, K-TIRADS, and C-TIRADS in Bethesda categories III/IV thyroid nodules. All Nodules **(A)**; Nodules ≤1cm **(B)**; Nodules >1 cm **(C)**.

Through an analysis of the receiver operating characteristic curve and based on the maximum Youden index, the optimal cutoff values for ACR-TIRADS, K-TIRADS, and C-TIRADS were determined to be 5, 5, and 4c, respectively (**Figure 1**). Regarding the identification of thyroid nodules, ACR-TIRADS classified 38 as benign and 42 as malignant, while K-TIRADS identified 44 as benign and 36 as malignant nodules. In contrast, C-TIRADS identified 36 as benign and 44 as malignant nodules. Among all the Bethesda III/IV nodules, the AUC values for ACR-TIRADS, K-TIRADS, and C-TIRADS were 0.782, 0.767, and 0.842, respectively. Notably, the AUC for C-TIRADS was significantly higher than those for ACR-TIRADS and K-TIRADS (all *P*<0.05) (**Tables 4**, **5**). Although ACR-TIRADS demonstrated the highest sensitivity and negative predictive value, C-TIRADS exhibited superior specificity, accuracy, and positive predictive value (**Table 6**).

**Table 4:** The area under the curve (AUC) of ACR-TIRADS, K-TIRADS, C-TIRADS.

	AUC	Standard Errora	Asymptotic P-value	Asymptotic 95% Confidence Interval	
				Lower Limit of 95% CI	Upper Limit of 95% CI
All Nodules					
ACR TI-RADS	0.782	0.053	0.000	0.678	0.885
K-TIRADS	0.767	0.053	0.000	0.663	0.872
C-TIRADS	0.842	0.045	0.000	0.754	0.930
Nodules ≤1 cm					
ACR TI-RADS	0.702	0.084	0.000	0.537	0.866
K-TIRADS	0.696	0.083	0.016	0.533	0.859
C-TIRADS	0.773	0.074	0.018	0.628	0.919
Nodules >1 cm					
ACR TI-RADS	0.857	0.065	0.000	0.731	0.984
K-TIRADS	0.828	0.071	0.000	0.689	0.966
C-TIRADS	0.916	0.047	0.000	0.823	1.008

**Table 5:** Comparison of AUC between ACR-TIRADS, K-TIRADS, and C-TIRADS.

	AUC difference	95% CI	Z value	P-value
All Nodules				
C-TIRADS VS. ACR-TIRADS	0.060	0.000–0.117	2.078	0.038
C-TIRADS VS. K-TIRADS	0.075	0.022–0.128	2.769	0.006
ACR-TIRADS VS.K -TIRADS	0.015	-0.037–0.066	0.548	0.583
Nodules ≤1 cm				
C-TIRADS VS. ACR-TIRADS	0.072	−0.009–0.152	1.752	0.080
C-TIRADS VS. K-TIRADS	0.077	−0.002–0.152	2.025	0.043
ACR-TIRADS VS. K-TIRADS	0.005	−0.069–0.080	0.143	0.886
Nodules >1 cm				
C-TIRADS VS. ACR-TIRADS	0.058	−0.021–0.138	1.443	0.149
C-TIRADS VS. K-TIRADS	0.088	0.010–0.165	2.209	0.027
ACR-TIRADS VS. K-TIRADS	0.029	−0.053–0.112	0.695	0.487

**Table 6:** Diagnostic value of ACR-TIRADS, K-TIRADS, C-TIRADS.

	Sensitivity (%)	Specificity (%)	Accuracy (%)	PPV (%)	NPV (%)
All Nodules					
ACR-TIRADS	78.05	74.36	76.25	80.00	76.32
K-TIRADS	68.29	79.49	75.00	77.78	70.45
C-TIRADS	73.17	84.62	78.75	83.33	75.00
Nodules ≤1 cm					
ACR-TIRADS	74.07	64.71	70.45	76.92	61.11
K-TIRADS	66.67	70.59	68.18	78.26	57.14
C-TIRADS	70.37	76.47	72.73	82.61	61.90
Nodules >1 cm					
ACR-TIRADS	85.71	81.82	83.33	75.00	90.00
K-TIRADS	71.43	86.36	80.56	76.92	80.61
C-TIRADS	78.57	90.91	86.11	84.62	86.96

### 3.5 Diagnostic value of the three TI-RADS for nodules of different sizes

Among the 36 Bethesda III/IV nodules >1 cm, 14 were benign, and 22 were malignant. The AUC values for ACR-, K-, and C-TIRADS were 0.857, 0.828, and 0.916, respectively. Among the 44 Bethesda III/IV nodules ≤1 cm, 17 were benign, and 27 were malignant, with AUC values of 0.702, 0.696, and 0.773 for ACR-, K-, and C-TIRADS, respectively. The AUC for C-TIRADS was significantly higher than that for K-TIRADS in both “nodules ≤1 cm” and “nodules >1 cm” groups (all *P*<0.05) (**Tables 4**, **5**).

## 4 Discussion

The TBSRTC categorizes indeterminate cytological results into three classes: III, IV, and V. Given the high ROM associated with class V nodules, TBSRTC recommends treating them as malignant, typically through subtotal thyroidectomy or lobectomy. Consequently, this study excluded Class V nodules and focused solely on Class III and IV. Although the Bethesda System standardizes the classification and terminology of thyroid cytopathology and provides recommended ROMs and clinical management strategies for each category, clinical decision-making for indeterminate cytological types remains a significant challenge for both clinicians and patients. Therefore, accurately estimating the ROM and distinguishing between benign and malignant lesions is crucial for determining appropriate treatment strategies. Understanding the predictive factors of malignancy in these nodules is of paramount importance.

Variability has been demonstrated in the malignancy rates of Bethesda category III and IV nodules (7). In our study, the malignancy rate for category III nodules was 50.7%, and that for category IV nodules was 55.6%. These rates exceed those recommended by the TBSRTC guidelines and align with findings from several other studies, which report malignancy rates ranging from 36% to 67% for Bethesda III and 40% to 83% for category IV nodules (8–10). However, a large cohort study reported significantly lower malignancy rates (25% for category III and 27.6% for category IV nodules) (11). This discrepancy may be attributed to our study’s exclusive inclusion of surgically confirmed cases, excluding those followed up clinically or subjected to repeated FNA. Additionally, there is a degree of subjectivity in the cytological diagnosis of Bethesda III/IV nodules, as interpretations can vary among cytopathologists. Consequently, the incidence of malignant nodules in this study was relatively high.

Our study examined the predictive value of clinical and ultrasound characteristics in distinguishing between benign and malignant nodules. The results indicated no significant correlation between age, sex, and pathological type (benign or malignant), which is consistent with previous findings (9, 12). However, the results related to thyroid-stimulating hormone are controversial. Our study suggests that thyroid-stimulating hormone levels are not associated with malignancy risk. In contrast, other studies indicated that higher serum thyroid-stimulating hormone levels are linked to an increased risk of thyroid cancer in cytologically indeterminate nodules, aiding in malignancy risk stratification (13, 14). Furthermore, our study identified statistically significant differences in ultrasound characteristics, such as internal composition, echogenicity, shape, margins, and echogenic foci, between benign and malignant nodules. Malignant nodules are more likely to exhibit ultrasound features, such as solid composition, hypoechogenicity or marked hypoechogenicity, ill-defined or irregular margins, lobulated shape, microcalcifications, and a taller-than-wide aspect ratio. This suggests that ultrasound characteristics have predictive value for assessing malignancy risk in Bethesda III/IV nodules. However, the number of these features varies significantly across different studies (7).

This study employed three TI-RADS classifications, each based on specific ultrasound features. The ACR-TIRADS estimates the malignancy risk of thyroid nodules through a total score. This system assigns a score to each ultrasound feature and sums these scores to determine the final classification of the nodule. Different total scores correspond to different risk categories. However, the scores assigned to each ultrasound feature are primarily based on expert opinion rather than statistical analysis (15). Therefore, the accuracy of ACR-TIRADS in predicting thyroid malignancies remains questionable. K-TIRADS is a pattern-based system, and its classification is determined by weighting different ultrasound features (16). Considering that the same ultrasound feature may exhibit different weights in various studies, this introduces a certain degree of uncertainty in the practical application of K-TIRADS. It is difficult to assume that K-TIRADS can appropriately weight all ultrasound features in a fully suitable manner. This uncertainty may affect its applicability and diagnostic performance across different populations (17, 18). Unlike ACR-TIRADS and K-TIRADS,C-TIRADS is established using a counting method. This method is considered more convenient and practical in clinical settings than complex weighting schemes (6). C-TIRADS determines the score for each ultrasound feature through statistical analysis, classifying nodules based on these scores. The core of the counting method lies in the simple enumeration of malignant features, rather than intricate weighting calculations (6). This simplification streamlines the operational process and reduces the potential for subjective judgment errors, thereby enhancing diagnostic consistency. Consequently, for Bethesda III/IV category nodules, C-TIRADS may offer more accurate risk stratification due to its detailed scoring system, potentially mitigating the risks of both overdiagnosis and underdiagnosis.

In this study, ACR-, K-, and C-TIRADS effectively stratified the risk of all nodules with AUC values of 0.782, 0.767, and 0.842, respectively. The optimal cutoff values were ACR-TIRADS category 5, K-TIRADS category 5, and C-TIRADS category 4C, indicating that all three TI-RADS classifications hold diagnostic value for nodules cytologically, using FNA, classified as Bethesda III/IV. Notably, C-TIRADS demonstrated superior diagnostic performance compared to ACR TI-RADS and K-TIRADS, as indicated by a significantly higher area under the curve (AUC) (P < 0.05). The sensitivities of the ACR-, K-, and C-TIRADS were 78.05%, 68.29%, and 73.17%; the specificities were 74.36%, 79.49%, and 84.62%; and the accuracies were 76.25%, 75.00%, and 78.75%, respectively. The positive predictive values were 80.00%, 77.78%, and 83.33%, and the negative predictive values were 76.32%, 70.45%, and 75.00%, respectively. The C-TIRADS exhibited the highest specificity, accuracy, and positive predictive value, whereas the ACR TI-RADS had the highest sensitivity and negative predictive value. Prior research has yielded similar findings. For instance, Mao et al. demonstrated that C-TIRADS exhibits superior performance in differentiating benign from malignant thyroid nodules compared to K-TIRADS and ACR-TIRADS (19). Corroborating this, Topcuoglu et al. (20), in a comparative analysis of six commonly used thyroid nodule diagnostic guidelines – ACR-TIRADS, Kwak-TIRADS, K-TIRADS, EU-TIRADS, American Thyroid Association (ATA) guidelines, and C-TIRADS– also found C-TIRADS to possess the optimal diagnostic performance in distinguishing between benign and malignant thyroid nodules, with a higher Area Under the Curve (AUC) than the other guidelines. This aligns closely with the results of the present study. However, the findings of Lin et al. (21) present a contrasting perspective, suggesting no significant difference in diagnostic efficacy among ACR-TIRADS, K-TIRADS, and C-TIRADS for nodules classified as atypia of undetermined significance/follicular lesion of undetermined significance (AUS/FLUS). This discrepancy may stem from heterogeneity in the study populations. While the present study primarily focused on Bethesda categories III and IV nodules, Lin et al.’s research specifically examined AUS/FLUS nodules. Consequently, C-TIRADS may demonstrate superior performance in the overall sample, whereas the efficacy differences among various guidelines may be less pronounced within specific subgroups. Future research should further investigate the diagnostic performance of different TI-RADS classification systems across various types of thyroid nodules. Additionally, our study validated the ROM for each category of the ACR-, K-, and C-TIRADS. The calculated malignancy rates for most categories fell within the guideline-recommended ranges, with the ROM increasing progressively with higher grading, and a high correlation was observed among the three TI-RADS. These findings were consistent with those of previous studies (10, 22). However, the ROM for ACR-TIRADS category 4 and C-TIRADS category 4A nodules exceeded the guideline-recommended rates. This discrepancy may be attributed to interobserver variability and the study’s focus on Bethesda III/IV nodules, whereas the ACR TI-RADS, K-TIRADS, and C-TIRADS encompassed all thyroid nodules.

To evaluate the diagnostic performance of these guidelines across different nodule sizes, we selected 1 cm as the cutoff value, which is the standard for the pathological diagnosis of microcarcinomas (23). Despite the relatively small sample sizes in each subgroup, we conducted a thorough analysis of the available data to provide valuable insights for clinical practice. The study revealed that the sensitivity, specificity, accuracy, and AUC of ACR-, K-, and C-TIRADS for nodules >1 cm were all higher compared with those for nodules ≤1 cm. This indicates that these systems have superior diagnostic performance for nodules measuring >1 cm in diameter. This finding aligns with the results of Li et al. (24), further confirming the significant impact of nodule size on the diagnostic performance of TI-RADS. This may be because larger nodules tend to exhibit clearer sonographic features (e.g., shape, margin, and internal structure) on ultrasound, thereby enhancing the diagnostic accuracy of TI-RADS. Conversely, smaller nodules may present with less distinct benign or malignant characteristics, increasing diagnostic difficulty and resulting in relatively lower performance of the three TI-RADS systems for these nodules. Among the three classification systems, C-TIRADS consistently exhibited the highest AUC for both ≤1 cm and >1 cm nodules. Although AUC is the key parameter for evaluating overall diagnostic validity, we contend that the high AUC of C-TIRADS in nodules of varying sizes is not solely attributable to its high specificity and accuracy. The relatively high negative predictive value and the highest positive predictive value also significantly enhance its performance. This indicates that although C-TIRADS may classify certain malignant nodules into lower categories, nodules categorized as C-TIRADS 4C and 5 are predominantly malignant. Consequently, this classification system effectively aids in distinguishing malignant nodules from benign ones.

This study has the following limitations:First, this study is a single-center retrospective study with a relatively small sample size. This may limit the generalizability of the study results and affect the reliability of subgroup analyses. Future studies should further validate the findings of this study with larger sample sizes to enhance the external validity and stability of the results. Second, the image analysis in this study is based on static images, and there may be differences in the understanding of TI-RADS classification among different physicians, which could lead to certain biases in classification. This subjectivity may affect the consistency and accuracy of the results. Third, this study only included nodules confirmed by surgical pathology, resulting in a relatively high incidence of malignant tumors, with papillary thyroid carcinoma (PTC) accounting for the majority of malignant nodules. This selection bias may limit the applicability of the study results to other types of thyroid cancer and requires further validation in a broader population.

In summary, the three TI-RADS classifications have diagnostic value for determining the benign or malignant nature of Bethesda III/IV nodules. The diagnostic performance of ACR-, K-, and C-TIRADS for nodules >1 cm was superior to that for nodules ≤1 cm. Regardless of the nodule size, C-TIRADS demonstrated a higher AUC compared with the other two TI-RADS classifications, suggesting that C-TIRADS may be more effective in evaluating and managing Bethesda III/IV nodules, particularly in Chinese populations.

## Data Availability

The original contributions presented in the study are included in the article/supplementary material. Further inquiries can be directed to the corresponding author.
